# Reappraising traumatic brain injury and ventilator-associated pneumonia through the brain-lung-immune-microbiome axis

**DOI:** 10.3389/fneur.2026.1844556

**Published:** 2026-07-16

**Authors:** Lielong Mao, Zhiwei Yang, Feijun Jiang

**Affiliations:** 1 Department of Respiratory and Critical Care Medicine, The Second Hospital of Zhuzhou, Zhuzhou, Hunan, China; 2 Department of Medical Affairs, The Second Hospital of Zhuzhou, Zhuzhou, Hunan, China; 3 Department of Scientific Research, The Second Hospital of Zhuzhou, Zhuzhou, Hunan, China

**Keywords:** brain-lung, immune, microbiome, traumatic brain injury, ventilator-associated pneumonia

## Abstract

Ventilator-associated pneumonia (VAP) remains the most prevalent and lethal infectious complication among patients with severe traumatic brain injury (TBI) requiring invasive mechanical ventilation. Historically, the pathogenesis of VAP within neurocritical care settings has been attributed to mechanical and physical factors. This traditional paradigm posits that the endotracheal tube bypasses natural upper airway defenses, impairs glottic reflexes, and allows for the continuous micro-aspiration of pathogen-laden oropharyngeal secretions into the lower respiratory tract. However, this biomechanical model fails to adequately explain the disproportionately high incidence of VAP in TBI patients compared to other critically ill populations. A profound conceptual paradigm shift is transitioning the focus from isolated airway mechanics toward multidimensional biological construct: the Brain-Lung-Immune-Microbiome Axis. The fundamental catalyst for respiratory vulnerability following neurotrauma is central nervous system injury-induced immunodepression syndrome (CIDS), a profound systemic immune remodeling driven by acute neuroendocrine and autonomic dysregulation, as well as the translocation of pathogenic gut microbiota to the lungs. By synthesizing evidence from neuroimmunology, microbiology, and critical care medicine, this narrative review details the tripartite interplay of neurological trauma, immune exhaustion, and microbiome evolution. Ultimately, this review evaluates emerging host-directed immunomodulatory therapies and microbiome-targeted interventions, advocating for a critical transition from simple mechanical airway hygiene to precision immuno-microbiome therapeutics in the neuro-intensive care unit.

## 1 Introduction

Traumatic brain injury (TBI) constitutes a severe global public health crisis, characterized by exceptionally high rates of mortality, prolonged hospitalization, and devastating long-term neurological disabilit y (1). In the acute phase of severe TBI, patient survival is highly dependent on admission to specialized neuro-intensive care units (neuro-ICUs), where invasive mechanical ventilation (IMV) is frequently initiated. Mechanical ventilation is a life-saving intervention utilized for airway protection in comatose patients, precise management of elevated intracranial pressure through controlled PaCO_2_, and the optimization of cerebral oxygenation (2). While IMV is indispensable, it paradoxically introduces significant iatrogenic risks, the most perilous of which is ventilator-associated pneumonia (VAP). Clinically, VAP is defined by the presence of new or progressive radiographic lung infiltrates accompanied by systemic signs of infection and purulent tracheobronchial secretions. The diagnosis is optimally confirmed by quantitative microbiological cultures of bronchoalveolar lavage fluid or endotracheal aspirates. It is crucial to distinguish between early-onset VAP (occurring within the first 4 days of intubation, typically caused by antimicrobial-susceptible community-acquired pathogens) and late-onset VAP (occurring after 5 days, frequently involving multidrug-resistant pathogens). Epidemiological data consistently demonstrate that patients with acute brain injuries exhibit an extraordinary and unique susceptibility to respiratory infections, with the incidence of VAP ranging alarmingly between 42% and 51% in mechanically ventilated TBI cohorts (3). Furthermore, the exceptionally high incidence of VAP in TBI cohorts cannot be solely attributed to immune mechanisms; it is heavily influenced by multiple clinical confounders. These include the depth of sedation, severely impaired gag reflexes that heighten micro-aspiration risk, overall injury severity scores, tracheostomy timing, prior empiric antibiotic exposure, and the variable adherence to preventive ventilator bundles across different intensive care units.

The traditional understanding of VAP pathogenesis has predominantly centered on exogenous, mechanical, and physical factors. The placement of an endotracheal tube artificially disrupts the anatomical barriers of the glottis and larynx, severely impairs the glottic reflex, and prevents effective coughing (1). Concurrently, prolonged supine positioning and the inevitable pooling of secretions above the endotracheal tube cuff facilitate the continuous micro-aspiration of contaminated bacteria from the upper gastrointestinal tract and oropharynx directly into the lower respiratory tract (4). Based on this mechanical understanding, global clinical guidelines have universally championed prophylactic “VAP bundles.” These bundles emphasize physical interventions such as strict head-of-bed elevation, continuous subglottic aspiration, and rigorous oral hygiene with chlorhexidine. While these standardized measures have successfully reduced the incidence of VAP in general surgical and medical ICU populations, their efficacy is markedly attenuated in the neurocritical care setting. This clinical discrepancy heavily implies that physical micro-aspiration is merely the proximal vector, rather than the fundamental etiology, of TBI-associated VAP.

Recent and rapid advancements in neuroimmunology, transcriptomics, and multi-omics sequencing have introduced a transformative conceptual framework. It is now understood that the systemic and local vulnerability of the lungs following neurological trauma is fundamentally governed by the Brain-Lung-Immune-Microbiome Axis. This axis posits that the central nervous system (CNS), the systemic immune system, and the host microbiota do not exist in isolation; rather, they engage in a delicate, continuous, and highly regulated homeostatic dialogue (5). Severe TBI violently disrupts this systemic dialogue, initiating a pathological cascade that is best conceptualized through the emerging “Triple-Hit Hypothesis” (2).

The primary hit involves the initial mechanical brain trauma, which instantly activates neurogenic inflammation and triggers a massive, uncoordinated, and sustained surge of the sympathetic nervous system and the hypothalamic-pituitary-adrenal (HPA) axis. This neuroendocrine storm induces a profound state of secondary, acquired immunodeficiency, formally termed CNS injury-induced immunosuppression syndrome (CIDS) (6). The secondary hit is mediated by the subsequent iatrogenic injury of positive-pressure mechanical ventilation and early infectious challenges. These secondary factors exploit the weakened systemic immune state to trigger local pulmonary inflammation, vascular permeability, and ventilator-induced lung injury (2). Finally, the tertiary hit encompasses the rapid, trauma-induced ecological collapse of the intestinal and respiratory microbiomes. Systemic immune dysregulation significantly compromises mucosal barriers, leading to increased intestinal permeability, the systemic translocation of gut pathogens to distant organs, and a precipitous decline in commensal respiratory flora. This ultimately culminates in the unchecked colonization of the lower airways by highly virulent, drug-resistant bacterial phenotypes (7).

Recognizing VAP as a downstream, end-stage manifestation of CIDS and systemic microbiome collapse—rather than an isolated, mechanically driven pulmonary event—fundamentally redefines the diagnostic and therapeutic landscape. The following sections will comprehensively dissect the molecular, cellular, and systemic mechanisms driving this axis. This narrative review will explore exactly how severe neural injury orchestrates immune failure, how the resulting physiological milieu dictates microbiome evolution, and how novel, targeted therapies can intercept this pathological cascade to improve outcomes in neurocritical care. This will provide new insights into the pathogenesis of VAP after TBI and provide feasible directions for controlling the disease. This will facilitate the development of subsequent mechanism experiments and the study of clinical cohorts.

This article is a narrative review. A comprehensive literature search was conducted using PubMed, Scopus, and Web of Science databases to identify relevant literature. Search terms included combinations of “traumatic brain injury,” “ventilator-associated pneumonia,” “CIDS,” “brain-lung axis,” “microbiome,” “dysbiosis,” “immunoparalysis,” and specific multidrug-resistant pathogens. Inclusion criteria encompassed peer-reviewed clinical trials, observational cohorts, murine models, and highly relevant mechanistic *in vitro* studies published in English. Articles focusing primarily on non-infectious acute respiratory distress syndrome (ARDS) without a neurotrauma component were excluded.

## 2 Brain injury-induced systemic immune remodeling: the pathogenesis of CIDS

For decades, the central nervous system was viewed through the lens of immune privilege, believed to be strictly shielded by the blood-brain barrier (BBB) and largely devoid of conventional lymphatic drainage. This archaic view has been entirely superseded by the recognition of complex, bidirectional, and highly sensitive neuro-immune communication networks. When the brain sustains a severe mechanical impact, the sudden disruption of the BBB allows for the immediate release of damage-associated molecular patterns (DAMPs), brain-specific autoantigens, and high concentrations of pro-inflammatory cytokines into the systemic circulation. This initial, hyper-inflammatory systemic response is rapidly and aggressively countered by a profound, centrally mediated compensatory anti-inflammatory response syndrome. The evolutionary intent of this response is theoretically neuroprotective; it is designed to suppress an overwhelming, self-destructive autoimmune attack against exposed, highly vulnerable neural tissues. However, the physiological cost of this necessary neuroprotection is CIDS, a systemic immune paralysis that renders the host completely defenseless against opportunistic nosocomial infections, with VAP being the most frequent and fatal manifestation (8).

Formally conceptualized by Meisel et al., CIDS is operationally defined as a centrally mediated, rapid, and sustained systemic immune deactivation following severe brain injury. Clinically, it is monitored through specific immune biomarkers, most notably a sustained drop in monocytic HLA-DR expression (often falling below the critical threshold of 30% positive monocytes or 8,000 antibodies per cell) and profound absolute lymphopenia. While CIDS shares phenotypic similarities with sepsis-induced immunoparalysis or generalized trauma-induced immune dysfunction—such as elevated anti-inflammatory cytokines and impaired phagocytosis—CIDS is distinctly characterized by its initiating trigger: a massive, central neurological insult and subsequent neuroendocrine storm, rather than peripheral pathogen-associated molecular patterns (PAMPs) or localized tissue destruction. The efferent arm of CIDS is intricately orchestrated by two primary, distinct, yet overlapping neuroendocrine pathways: the HPA axis and the autonomic nervous system (ANS).

### 2.1 The HPA axis overdrive

The profound physiological stress of a traumatic brain injury immediately activates the paraventricular nucleus of the hypothalamus, prompting the rapid release of corticotropin-releasing hormone (CRH). This biochemical signal traverses the hypophyseal portal system, stimulating the anterior pituitary gland to secrete massive volumes of adrenocorticotropic hormone (ACTH). ACTH subsequently circulates systemically, driving the adrenal cortex to synthesize and release extraordinary quantities of glucocorticoids, predominantly cortisol, into the bloodstream. Acute and sustained activation of the HPA axis induces profound, broad-spectrum anti-inflammatory and immunosuppressive effects across virtually all innate and adaptive immune cell lineages.

Elevated circulating cortisol exerts its immunosuppressive effects through a combination of genomic and non-genomic mechanisms. Cortisol easily permeates the lipid bilayer of circulating leukocytes, binding to cytosolic glucocorticoid receptors with high affinity. This newly formed hormone-receptor complex rapidly translocates to the cell nucleus, where it directly acts as a transcription factor. It actively inhibits the transcription of vital pro-inflammatory cytokines, notably Interleukin-1 beta (IL-1β), Tumor Necrosis Factor-alpha (TNF-α), and Interleukin-6 (IL-6), while simultaneously upregulating the expression of potent anti-inflammatory mediators such as Interleukin-10 (IL-10) and Transforming Growth Factor-beta (TGF-β) (9).

Furthermore, high systemic cortisol levels actively restrict the physical migration and proliferative capacity of peripheral T lymphocytes. Experimental murine models of mild to severe TBI demonstrate that plasma cortisol spikes exponentially within the first 4 h post-injury. This spike corresponds perfectly with a significant, measurable decrease in peripheral T-cell numbers and profoundly impaired *ex vivo* T-cell migration (10). This migratory inhibition is heavily mediated by the suppression of intracellular cyclic adenosine monophosphate (cAMP) levels within T-cells. The administration of phosphodiesterase inhibitors, such as rolipram, which artificially elevate and sustain intracellular cAMP, has been shown to completely reverse glucocorticoid-induced T-cell arrest. This explicitly underscores the precise biochemical etiology of this specific facet of immunosuppression, linking central hormone release directly to peripheral cellular paralysis.

### 2.2 Autonomic nervous system dysregulation and lymphoid apoptosis

While the HPA axis acts broadly via systemic humoral signaling, the ANS provides direct, hardwired neural control over the peripheral immune organs. The ANS is divided into the sympathetic nervous system (SNS) and the parasympathetic nervous system (PNS), which directly innervate primary and secondary lymphoid organs, including the bone marrow, thymus, lymph nodes, and spleen (11).

Severe TBI frequently results in a catastrophic loss of cortical inhibition over the brainstem and subcortical sympathetic nuclei. This loss of regulatory oversight leads to an uncontrolled, massive outpouring of adrenergic energy, clinically manifested as paroxysmal sympathetic hyperactivity (PSH) or a “sympathetic storm” (12–14). This sympathetic overdrive results in a systemic release of catecholamines, primarily norepinephrine and epinephrine, from both the adrenal medulla and direct sympathetic nerve terminals. The spleen, serving as a critical reservoir for lymphocytes, monocytes, and mature immune cells, receives exceptionally dense sympathetic innervation. Following neurotrauma, the excessive release of norepinephrine from splenic sympathetic nerve terminals binds directly to β2-adrenergic receptors (β2ARs) highly expressed on the surface of splenic B and T lymphocytes.

The continuous, unyielding stimulation of β2ARs by post-injury catecholamine surges initiates a lethal intracellular cascade within these immune cells. Intense β2AR activation directly upregulates the expression of the pro-apoptotic protein Bim (Bcl2-Interacting Mediator of Cell Death). The rapid intracellular accumulation of Bim neutralizes protective, anti-apoptotic Bcl-2 proteins. This leads to mitochondrial membrane permeabilization, the subsequent release of cytochrome c into the cytosol, and the ultimate activation of Caspase-3, the executioner enzyme of the apoptotic pathway (15). This massive, catecholamine-driven apoptosis leads to rapid splenic atrophy, profound systemic lymphopenia, and a drastic reduction in the absolute number of circulating immunocompetent cells available to fight respiratory infections. Although this research focuses on spinal cord injury, it also offers some insights for TBI. *In vivo* studies utilizing high-level spinal cord injury models have confirmed that the simultaneous pharmacological antagonism of glucocorticoid receptors and β2-adrenergic receptors can significantly diminish lymphocyte Bim levels and completely reverse injury-induced splenic lymphopenia. Initial evidence linking catecholamines directly to lymphocyte apoptosis via Bim upregulation was established in high-level spinal cord injury models. However, this critical mechanism has been directly corroborated in central nervous system injury models more directly analogous to TBI, where sympathetically mediated lymphoid atrophy and functional immune deactivation were robustly demonstrated. This suggests that the destructive synergy between the HPA and SNS axes is the primary molecular driver of CIDS.

**Figure 1:**
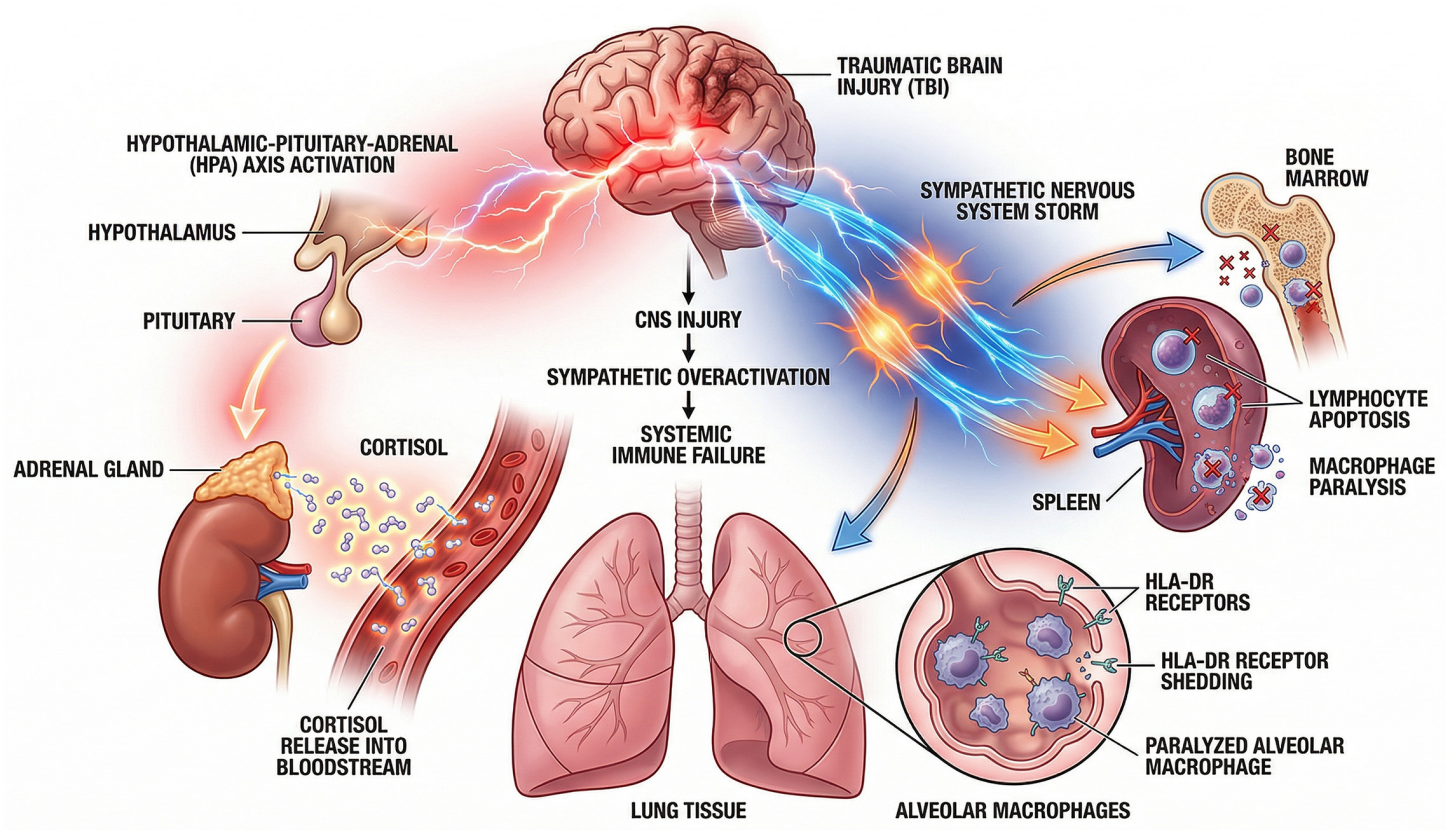
Macro-mechanism of central nervous system injury-induced immunosuppression syndrome. Severe traumatic brain injury triggers a massive neuroendocrine storm, activating the hypothalamic-pituitary-adrenal (HPA) axis and the sympathetic nervous system. The HPA axis overdrive leads to excessive cortisol release, while the sympathetic storm induces the apoptosis of splenic lymphocytes via β2-adrenergic receptor-mediated pathways. Concurrently, these systemic signals cause the epigenetic paralysis of lung-resident alveolar macrophages, characterized by a profound downregulation of surface HLA-DR expression, ultimately leading to a collapse of the local pulmonary immune barrier.

Simultaneously, the parasympathetic nervous system contributes to this immune remodeling via the cholinergic anti-inflammatory pathway. Efferent signals traveling through the vagus nerve stimulate the release of acetylcholine, which binds to α7-nicotinic acetylcholine receptors (α7nAChR) heavily expressed on the surface of tissue macrophages. While this pathway normally functions to prevent runaway inflammation by inhibiting the nuclear translocation of NF-κB, in the context of TBI, its overactivation acts synergistically with sympathetic apoptosis to force surviving macrophages into a deeply deactivated, tolerogenic state, severely compromising innate immune responses (Figure 1, Table 1).

**Table 1:** Neuroendocrine and autonomic pathways driving CNS injury-induced immunosuppression syndrome (CIDS).

Autonomic/endocrine pathway	Primary systemic mediator	Target cellular receptors	Intracellular signaling mechanism	Ultimate immunological consequence
**HPA axis (endocrine)**	Cortisol/glucocorticoids	Cytosolic glucocorticoid receptors	Decreased intracellular cAMP, inhibition of NF-κB gene transcription	Decreased T-cell migration, profound suppression of Th1 cytokines, enhancement of Th2 cytokines
**Sympathetic nervous system**	Norepinephrine/epinephrine	β2-adrenergic receptors (β2AR)	Upregulation of pro-apoptotic Bim protein, subsequent Caspase-3 activation	Splenic and bone marrow lymphopenia, widespread T and B lymphocyte apoptosis
**Parasympathetic nervous system**	Acetylcholine	α7-nicotinic acetylcholine receptor (α7nAChR)	Inhibition of NF-κB translocation in macrophages	Cholinergic anti-inflammatory pathway overactivation, exacerbating macrophage phagocytic failure

### 2.3 Defining the scope of CIDS: TBI, CNS injuries, and critical illness

It is crucial to emphasize that while this review focuses on the clinical context of TBI, CIDS is not a TBI-specific phenomenon. The neuroendocrine mechanisms described—namely the paroxysmal sympathetic storm and HPA axis overdrive—are conserved physiological responses common to various forms of severe acute central nervous system insults, including large hemispheric stroke, spontaneous intracerebral hemorrhage, and high-level spinal cord injury. Therefore, the resulting immune remodeling in TBI shares a fundamental pathogenic pathway with other severe CNS injuries.

Furthermore, CIDS must be clinically distinguished from the generalized immunoparalysis observed in non-neurological critical illnesses, such as severe sepsis or polytrauma. Phenotypically, these syndromes overlap significantly, converging on profound lymphopenia, macrophage deactivation, and mHLA-DR downregulation. However, they diverge fundamentally in their initiating triggers. Sepsis-induced immunoparalysis is driven “bottom-up” by systemic exposure to peripheral PAMPs and massive systemic inflammatory response syndrome. In stark contrast, CIDS is triggered “top-down.” It is a centrally mediated, neurogenic phenomenon where the brain, isolated from initial peripheral infection, actively paralyzes an otherwise intact peripheral immune system via hardwired autonomic and endocrine signaling. Understanding this distinct central etiology is paramount for developing precise immunomodulatory strategies in the neuro-ICU.

## 3 Collapse of the pulmonary local immune barrier

The lungs represent the largest and most delicate mucosal surface area exposed continuously to the external environment. In healthy individuals, the pulmonary compartment maintains near-sterility through a highly coordinated, dual-layered defense mechanism: the innate cellular immunity orchestrated primarily by alveolar macrophages, and the mechanical clearance driven by the airway ciliated epithelium. In the context of the Brain-Lung-Immune Axis, CIDS severely impairs both layers, paving the ultimate path for VAP.

### 3.1 Epigenetic paralysis and HLA-DR downregulation in alveolar macrophages

Alveolar macrophages (AMs) are the sentinel, tissue-resident phagocytes of the lower respiratory tract. They are solely responsible for clearing inhaled microbes, effectively presenting antigens to memory T-cells, and maintaining overall local immune homeostasis without triggering unnecessary tissue-damaging inflammation (16). Following a severe TBI, the systemic barrage of catecholamines, glucocorticoids, and circulating DAMPs induces a state of deep, intractable “immunoparalysis” in these tissue-resident macrophages.

The most critical, universally recognized molecular signature of this paralysis is the severe downregulation of Human Leukocyte Antigen—DR isotype (HLA-DR) expression on the surface of circulating monocytes and lung-resident alveolar macrophages. HLA-DR is a major histocompatibility complex (MHC) class II cell surface receptor that presents processed peptide antigens directly to the T-cell receptor (TCR) on CD4+ helper T-cells. A high density of cell-surface HLA-DR is an absolute biological prerequisite for the initiation of an effective, targeted adaptive immune response. In healthy physiological states, over 90% of alveolar macrophages strongly express HLA-DR, constantly surveying the alveoli for foreign antigens. However, in the post-aggressive immunosuppression state uniquely induced by TBI, mHLA-DR expression plummets drastically (17). The fundamental paradigm of monocyte deactivation via mHLA-DR suppression and its subsequent reversal by exogenous IFN-γ was originally established in the landmark work by Docke et al. (18). This mechanistic pathway was later validated in humans, where clinical data definitively linked sustained mHLA-DR suppression to a significantly heightened risk of nosocomial infections in critically ill patient populations (19). Without sufficient HLA-DR molecules, alveolar macrophages completely fail to present bacterial antigens, leaving the adaptive immune system compromised to the colonizing opportunistic pathogens multiplying in the alveoli.

Furthermore, recent advanced transcriptomic and metabolomic profiling in critically ill patients not specific to TBI has revealed that severe systemic inflammation followed immediately by deep immunosuppression induces long-lasting epigenetic modifications in the alveolar macrophage pool. This “epigenetic scarring” physically alters chromatin accessibility, locking the macrophages into a deactivated, tolerogenic state. This state is characterized by drastically impaired phagocytic capacity, a severely reduced oxidative burst, and a profound inability to secrete requisite pro-inflammatory cytokines such as IL-12 and TNF-α upon secondary bacterial challenge. However, this data is derived from the non-TBI ICU patient population and is only used as one basis for extrapolation to TBI patients. Notably, metabolomic analysis of bronchoalveolar lavage fluid from TBI patients who develop VAP reveals distinct shifts in the pentose phosphate and citric acid cycle pathways, indicating a forced transition to anaerobic cellular metabolism in the lungs. Even when the initial physical brain injury stabilizes, this cellular epigenetic paralysis persists, eloquently explaining the delayed but relentless onset of late-stage VAP in neurocritical care patients.

### 3.2 Autonomic dysregulation of mucociliary clearance

If alveolar macrophages form the indispensable cellular defense, mucociliary clearance constitutes the primary mechanical defense mechanism of the conducting airways. The respiratory epithelium is extensively lined with ciliated cells covered by a biphasic layer: a highly regulated periciliary fluid layer and a viscoelastic mucus gel layer that physically traps inhaled pathogens (20, 21). The coordinated, rhythmic beating of millions of cilia propels this pathogen-laden mucus upward toward the pharynx, where it is either swallowed or expectorated.

Crucially, the ciliary beat frequency and local mucus rheology are not autonomous; they are tightly regulated by the autonomic nervous system. The airway epithelium contains rich, functional networks of autonomic receptors. Under physiological conditions, the baseline stimulation of mucosal and serosal β2-adrenergic receptors (via steady sympathetic tone) and muscarinic receptors (via parasympathetic vagal tone) act synchronously to upregulate intracellular calcium (Ca^2+^) and cAMP, directly stimulating ciliary beat frequency and ensuring continuous clearance (22).

However, following a TBI, the pathological sympathetic storm violently uncouples this delicate regulatory mechanism. The massive, sustained surge in circulating catecholamines causes a rapid desensitization and prolonged down-regulation of β2-adrenergic receptors on the respiratory epithelium, resulting in a paradoxical and complete failure to sustain ciliary motility. Furthermore, sympathetic overdrive dramatically alters systemic and pulmonary hemodynamics, drastically increasing pulmonary capillary hydrostatic pressure and inducing varying degrees of neurogenic pulmonary edema (NPE).

The resulting leakage of protein-rich fluid from the vasculature into the alveoli and airways triggers an immediate influx of neutrophils and the local release of highly destructive inflammatory cytokines, most notably TNF-α (23). High concentrations of local TNF-α, acting in conjunction with the overactivation of matrix metalloproteinases (specifically MMP-9), severely damage the structural integrity of the delicate epithelial barrier. Elevated MMP-9 degrades vital cell-cell and cell-matrix adhesions, leading to the direct sloughing, death, and physical loss of ciliated cells from the airway surface (with experimental models demonstrating a 25% loss of ciliated cells following intense inflammatory insults) (24). Simultaneously, the inflammatory milieu forces airway goblet cells and submucosal glands to increase the secretion of high-molecular-weight mucins, exponentially increasing mucus viscosity. The devastating confluence of reduced ciliary beat frequency (due to autonomic receptor desensitization), the physical loss of ciliated cells (due to MMP-9 mediated apoptosis), and highly viscous mucus results in total mucociliary stasis. Pathogens introduced via micro-aspiration are no longer cleared; instead, they are granted a stable, warm, nutrient-rich scaffold deep within the bronchial tree to colonize and proliferate.

## 4 Lung microbiome evolution, gut translocation, and inter-kingdom signaling

While the healthy lower respiratory tract was historically considered a sterile environment, modern sequencing reveals it harbors a low-biomass microbial community, though its resident versus transient nature remains debated. Modern culture-independent genomic techniques, notably 16S rRNA gene sequencing, have unequivocally debunked this, revealing that the healthy lower respiratory tract actually harbors a low-biomass, dynamically balanced microbiome (predominantly composed of phyla such as *Bacteroidetes* and *Firmicutes*) (25). This baseline state of eubiosis is maintained through a delicate, continuous balance of micro-aspiration of flora from the oral cavity, constant microbial elimination via functional mucociliary clearance, and the strict regulatory oversight of the host immune system (26). In the severely injured TBI patient, the combination of CIDS, mucociliary stasis, and the physical presence of an endotracheal tube precipitates a profound ecological shift of the respiratory microbiome, termed dysbiosis. Of course, this microbiome alteration is also driven by antibiotics, enteral nutrition, and ICU exposure.

### 4.1 The translocation of the gut-lung microbiome

The brain-lung axis cannot be accurately evaluated in isolation from the gastrointestinal tract. The advanced “Triple-Hit” hypothesis explicitly recognizes the gut microbiota as a critical intermediary vector in the pathogenesis of lung infection following brain trauma (27). Severe brain injury induces rapid, profound gastrointestinal hypomotility, splanchnic ischemia, and intense intestinal mucosal inflammation. This acute, systemic stress directly and severely impacts Paneth cells, specialized epithelial cells located in the crypts of the small intestine. Paneth cells are responsible for secreting an array of antimicrobial peptides that maintain the stability and composition of the enteric flora. TBI significantly reduces Paneth cell function and drives them toward apoptosis, leading to a catastrophic drop in the concentration of enteric antimicrobial peptides (28).

This localized failure of chemical defense permits the rapid, unchecked overgrowth of pathogenic, opportunistic bacteria within the gut lumen, particularly facultative anaerobes such as *Enterobacteriaceae* (29). Concurrently, the systemic inflammatory response severely compromises the tight junctions of the intestinal epithelium, creating the well-documented phenomenon of a “leaky gut” (30). The massively overgrown pathogenic bacteria and their highly inflammatory byproducts (such as lipopolysaccharide, LPS) translocate directly across the compromised intestinal barrier, enter the mesenteric lymph nodes, and are swept into the portal and systemic circulation, eventually seeding the pulmonary capillary bed from within the body.

Advanced microbial source-tracking techniques in murine models have provided compelling evidence of this gut-lung translocation. In specialized murine models of severe TBI, researchers utilized *SourceTracker* bioinformatics algorithms to demonstrate that while the lung microbiota of healthy, uninjured subjects contains 0% gut-derived sequences, by 7 days post-injury, nearly 50% (49.69%) of the entire lung tissue microbiota is directly sourced from the gut, which was the conclusion drawn in a mouse model of experimental severe TBI instead of human (28). This unambiguously indicates that the lungs of TBI patients are being continuously and heavily seeded by virulent gut bacteria originating from the bloodstream—a pathogenic vector that is entirely immune to mechanical oral care, chlorhexidine washes, or airway aspiration bundles.

### 4.2 Oropharyngeal down-migration and respiratory dysbiosis

Simultaneously, the exogenous vector of microbiome shift is relentlessly facilitated by the endotracheal tube (ETT). The ETT acts as a synthetic, physical highway bypassing the protective glottis, allowing the continuous, albeit microscopic, descent of highly altered oropharyngeal flora. In mechanically ventilated neurocritical patients, the rich, diverse community of normal oral commensals (such as health-associated *Corynebacterium*) is rapidly eradicated and replaced. Longitudinal metataxonomic analyses of endotracheal aspirates and bronchoalveolar lavage (BAL) fluid from TBI patients reveal a highly distinct temporal transition. Patients who ultimately progress to clinical VAP demonstrate a precipitous drop in microbial α-diversity (both richness and evenness), which is intimately accompanied by an explosive, monolithic overgrowth of pathogenic genera, primarily *Acinetobacter, Pseudomonas, Staphylococcus*, and *Escherichia-Shigella*(31). This stark dysbiosis correlates directly and proportionately with higher pro-inflammatory cytokine loads (IFN-γ and TNF-α) detected in the lung effluent and progressively worse oxygenation parameters (measured by PaO2/FiO2 ratios) (32).

### 4.3 Host-pathogen inter-kingdom signaling and biofilm formation

A critical mechanism at the intersection of microbiology and neuroendocrinology is that these opportunistic pathogens do not merely exploit the weakened host immune system passively; they actively sense, interpret, and respond to the host's neurological distress through an evolutionary mechanism known as “inter-kingdom signaling” (33).

Gram-negative ESKAPE pathogens, specifically *Acinetobacter baumannii* and *Pseudomonas aeruginosa*, represent the most recalcitrant, deadly, and multidrug-resistant etiologies of VAP globally, particularly in TBI cohorts. The extreme virulence of these specific pathogens is intrinsically linked to their ability to construct complex, highly resilient extracellular polymeric substance (EPS) biofilms on both the abiotic surface of the endotracheal tube and the biotic surface of the severely injured respiratory epithelium (34). Once established, biofilms confer near-total resistance to host macrophage phagocytosis and render standard intravenous antibiotic therapy largely ineffective.

Remarkably *in vitro* evidence, these bacteria have evolved dedicated, highly sensitive membrane sensor kinases that specifically detect human host catecholamines—the exact stress hormones surging at unprecedented levels in the bloodstream of a TBI patient. For example, in *P. aeruginosa*, the membrane sensor protein *RetS* directly intercepts host norepinephrine. This interaction immediately manipulates the bacterial *GacS/GacA* two-component regulatory system, drastically upregulating the transcription of critical quorum-sensing molecules and virulence factors. Furthermore, norepinephrine exposure aggressively accelerates the temperature-induced production of Pel (pellicle) and Psl (polysaccharide synthesis locus) exopolysaccharides, which are the fundamental structural building blocks required for robust biofilm architecture (35, 36).

Similarly *in vitro* evidence, in *A. baumannii*, host norepinephrine directly stimulates rapid bacterial proliferation and massively upregulates the expression of the *Csu* operon (*Csuab-A-B-C-D-E*). The *Csu* operon, masterfully regulated by the *BfmRS* two-component signaling system, governs the assembly of type I chaperone-usher pili (37). These specialized pili are absolutely critical for the initial adhesion of the bacteria to human alveolar epithelial cells and the subsequent maturation of complex 3D biofilm structures (38). Furthermore, the presence of host norepinephrine actively enhances the acquisition of host iron by the bacteria, utilizing the catechol ring of the hormone as a pseudo-siderophore, successfully overcoming host nutritional immunity barriers (39).

**Figure 2:**
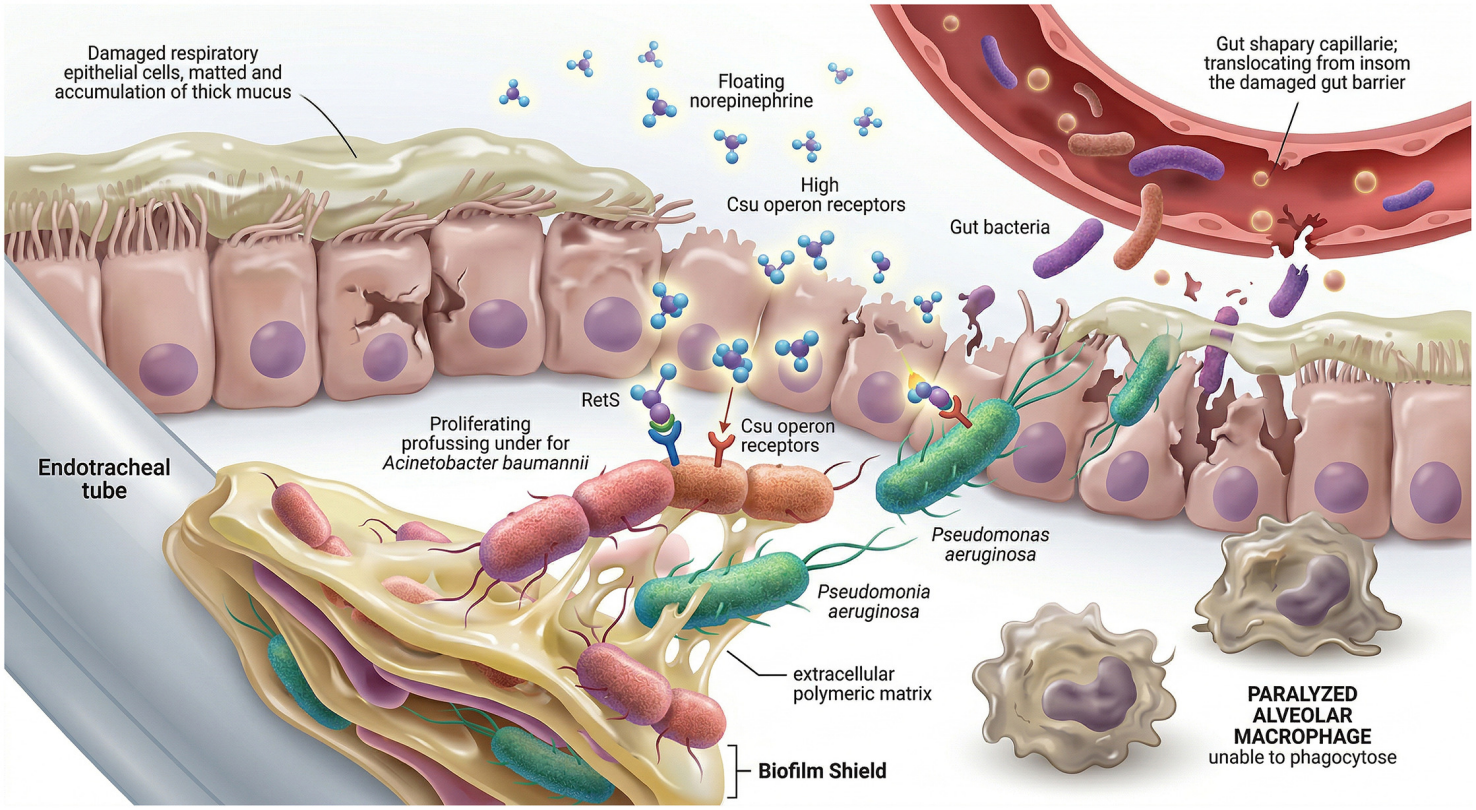
Pulmonary microbiome dysbiosis, gut translocation, and host-pathogen inter-kingdom signaling. The injured pulmonary microenvironment promotes dysbiosis and the formation of pathogenic biofilms. Dysregulation of the gut-lung axis leads to the systemic translocation of opportunistic enteric bacteria into the pulmonary capillary bed. Simultaneously, host stress hormones, such as norepinephrine, are actively detected by virulent pathogens (e.g., Pseudomonas aeruginosa and Acinetobacter baumannii) through specific membrane sensors like RetS. This inter-kingdom signaling directly stimulates the rapid construction of robust EPS biofilms on the injured respiratory epithelium and endotracheal tubes.

Therefore, the paroxysmal sympathetic storm of the TBI patient not only paralyzes the host's leukocyte defenses but acts as a direct, highly potent chemical catalyst for the most dangerous phase of bacterial pathogenesis, elegantly highlighting exactly why traditional, broad-spectrum antibiotic regimens frequently fail to eradicate biofilm-embedded VAP in neurocritical care environments (Figure 2, Table 2).

**Table 2:** Mechanisms of inter-kingdom signaling and biofilm formation in VAP-associated pathogens.

Pathogen	Associated VAP virulence mechanism	Host hormone trigger	Specific receptor/operon targeted	Resulting bacterial phenotype
Pseudomonas aeruginosa	Robust biofilm EPS production	Norepinephrine/epinephrine	*RetS* membrane sensor/*GacS/GacA* regulatory system	Upregulation of Pel/Psl polysaccharides, massive quorum sensing activation
**Acinetobacter baumannii**	Epithelial adhesion & proliferation	Norepinephrine	*BfmRS* two-component system/*Csu* Operon	Overexpression of type I chaperone-usher pili, enhanced iron acquisition (pseudo-siderophore)

## 5 Targeted brain-lung-immune-microbiome intervention strategies

Recognizing VAP as a terminal consequence of the dysregulated Brain-Lung-Immune-Microbiome Axis demands a sweeping therapeutic pivot. Prevention and treatment algorithms must evolve significantly beyond purely mechanical measures (such as routine suctioning, positioning, and cuff pressure monitoring) to directly counteract systemic immunoparalysis and proactively restore microbial eubiosis. Although the various measures mentioned below have some innovative potential, there is not sufficient evidence that there is a possibility that they do not target TBI-related VAP, so these measures are not ready for clinical trials, and further basic theoretical research is still needed.

### 5.1 Beta-adrenergic blockade

Given the mechanistic centrality of catecholamine excess in driving CIDS, the pharmacological attenuation of the sympathetic storm presents a logical therapeutic avenue. The administration of beta-adrenergic antagonists, such as propranolol, has garnered increasing support in TBI-specific literature. By competitively blocking β2-adrenergic receptors on splenic and circulating lymphocytes, beta-blockers actively interrupt the intracellular cascade leading to Bim-mediated apoptosis. Emerging clinical observations suggest that early, targeted beta-blockade in severe TBI not only blunts the dangerous systemic hyperadrenergic state but also preserves the peripheral lymphocyte pool, potentially mitigating the downstream risk of infectious complications.

### 5.2 Host-directed immunomodulatory therapies

The primary objective of targeted immunomodulation in severe TBI is to pharmacologically reverse CIDS, safely reactivating the paralyzed components of both innate and adaptive immunity before the dysbiotic microbiome can firmly establish impenetrable pathogenic biofilms.

#### 5.2.1 Interferon-gamma (IFN-γ)

Given that the pathognomonic and most reliable feature of alveolar macrophage and monocyte paralysis is the severe downregulation of surface HLA-DR, the exogenous administration of recombinant human Interferon-gamma (IFN-γ) presents a highly rational, mechanistic intervention. IFN-γ is a potent, naturally occurring pleiotropic cytokine that directly stimulates and upregulates MHC class II molecule expression on antigen-presenting cells, thereby restoring their critical ability to initiate T-cell responses and recognize invading pathogens. In small, highly targeted clinical case series involving patients with severe viral pneumonia and recurrent VAP demonstrating profound mHLA-DR depression, the subcutaneous administration of IFN-γ successfully restored mHLA-DR expression to near-normal levels, significantly increased the proportion of activated monocytes, and improved overall lung defense (40).

However, translating this to a broader ICU population has proven difficult. Larger multi-center randomized controlled trials evaluating IFN-γ for the blanket prevention of hospital-acquired pneumonia in unselected, mechanically ventilated critically ill patients have yielded cautionary results. A recent prominent European trial was terminated early by its data safety monitoring board, as routine IFN-γ failed to reduce the incidence of pneumonia and actually trended toward harm in the general cohort (41). The critical, nuanced insight gained here is the absolute necessity of rigorous patient phenotyping: indiscriminate immune stimulation in critical illness can inadvertently exacerbate latent, unrecognized hyper-inflammatory states. IFN-γ therapy holds substantial clinical promise but must be utilized strictly within a precision-medicine framework. It should be explicitly restricted to neuro-ICU patients who exhibit confirmed, persistent mHLA-DR suppression, continuously verified via real-time flow cytometry monitoring.

#### 5.2.2 Thymosin alpha-1 (Tα1)

Thymosin alpha-1 is a 28-amino acid acidic polypeptide naturally secreted by the human thymus gland. It functions as a powerful biological response modifier, actively promoting the maturation, differentiation, and survival of T-cells, effectively counteracting the massive T-cell apoptosis driven by the sympathetic-HPA axis described earlier (42). In rigorous clinical trials involving mechanically ventilated patients, the systemic administration of Tα1 significantly improved vital cellular immunity markers. Patients receiving the Tα1 intervention experienced a significant delay in the onset of early VAP and a marked reduction in both the total duration of mechanical ventilation and ICU length of stay (43).

#### 5.2.3 Interleukin-7 (IL-7)

IL-7 is a specific cytokine critical for the survival, proliferation, and expansion of the lymphoid lineage. Clinical evaluations utilizing compassionate-use IL-7 in severely immunosuppressed ICU patients have demonstrated that systemic IL-7 injections rapidly and safely reverse absolute lymphopenia, drastically improving both total CD4+ T-cell and Natural Killer (NK) cell counts (44). Importantly, extensive monitoring shows that IL-7 achieves this remarkable lymphoid restoration without provoking an aberrant, dangerous surge in pro-inflammatory cytokines (such as IL-6 or TNF-α), thereby successfully avoiding the inadvertent induction of a secondary, fatal cytokine storm. By successfully rescuing the peripheral lymphocyte pool from catecholamine-induced apoptosis, IL-7 restores the necessary adaptive immune response required to identify and clear persistent bacterial colonization deep within the lung tissue.

### 5.3 Microbiome-targeted modulations

Counteracting the massive, systemic shift in both gut and respiratory microbiomes represents the second, equally vital arm of a comprehensive, axis-targeted therapeutic strategy.

#### 5.3.1 Probiotic, prebiotic, and symbiotic therapies

To effectively combat the gut-to-lung translocation of pathogens driven by TBI-induced intestinal dysbiosis, the rapid restoration of enteric flora is paramount. Probiotics act locally within the gut lumen to competitively exclude pathogenic *Enterobacteriaceae* from binding to the epithelium, significantly enhance the structural integrity of the intestinal epithelial tight junctions to reverse “leaky gut,” and heavily stimulate the local production of mucosal IgA and potent anti-inflammatory short-chain fatty acids.

Extensive umbrella reviews and meta-analyses spanning dozens of randomized controlled trials and tens of thousands of mechanically ventilated, critically ill patients suggest a highly pronounced, reproducible benefit of prophylactic probiotic administration. Pooled statistical data conclusively indicate that probiotics confer an approximate 26% to 33% relative risk reduction in the overall incidence of VAP (45). Furthermore, probiotics are consistently associated with modest but highly statistically significant reductions in the duration of mechanical ventilation (by approximately 1.6 days), overall ICU length of stay, and the total duration of empiric antibiotic use (46).

However, translating these highly promising findings into universal, unalterable neurocritical care protocols is challenged by profound study heterogeneity. The clinical efficacy of probiotics is highly strain-specific, and the severely compromised gastrointestinal motility frequently observed in the acute TBI patient may severely impair the proper transit and mucosal engraftment of enterically administered flora (47, 48). Despite these specific limitations, the robust biological rationale and exceptional safety profile make microbiome restoration via precision probiotics a highly compelling, necessary adjunctive strategy for preventing the tertiary “hit” of dysbiosis-driven VAP.

#### 5.3.2 Selective digestive decontamination (SDD) and targeted prophylaxis

Recognizing that the highly dysbiosis oropharynx and stomach serve as the primary, immediate reservoirs for VAP pathogens, SDD protocols have been developed (49). SDD involves the routine, prophylactic application of non-absorbable topical antimicrobials to the oral cavity and gastrointestinal tract, frequently combined with a very short, targeted course of systemic intravenous antibiotics immediately upon intubation (50). By proactively eradicating the monolithic overgrowth of *Acinetobacter* and *Pseudomonas* before they can be micro-aspirated and form recalcitrant, mature biofilms in the lung, SDD targets the dysbiotic vector directly at its source. Extensive meta-analyses and trials specifically in severe neurological injury cohorts indicate that early prophylactic intravenous antibiotics (such as narrow-spectrum cephalosporins) significantly reduce the incidence of microbiologically confirmed early-onset pneumonia (51). While undeniably controversial due to the theoretical, long-term risk of accelerating systemic antimicrobial resistance across the ICU, SDD underscores the critical physiological principle that the preemptive, aggressive control of the host microbiome burden can dramatically mitigate the devastating infectious consequences of severe brain injury (Table 3).

**Table 3:** Targeted immune-microbiome intervention strategies in severe traumatic brain injury.

Intervention strategy	Target within the brain-lung axis	Primary mechanism of action	Current clinical status	Evidence level	TBI-specific	Key safety concerns	potential benefit
**Interferon-γ** **(IFN-γ)**	Pulmonary macrophage paralysis (CIDS)	Reverses epigenetic paralysis; upregulates mHLA-DR expression for vital antigen presentation	Mixed results in unselected patients; phase 2 RCT terminated early. Strictly requires precision phenotyping via flow cytometry prior to use	Clinical case series, Phase 2 RCTs	No (Extrapolated from unselected ICU/severe viral pneumonia)	Risk of exacerbating active neuroinflammation and worsening secondary brain injury if given indiscriminately	Restores mHLA-DR expression, increases activated monocytes, and improves overall lung defense
**Thymosin alpha-1 (Tα1)**	Systemic lymphopenia (CIDS)	Rescues T-cells from catecholamine apoptosis; improves CD4+/CD8+ ratios and promotes lymphoid maturation	Utilized in clinical trials for mechanically ventilated critically ill patients	RCTs	No (Mechanically ventilated ICU populations)	Generally well-tolerated; potential risk of overstimulating immune response requires standard monitoring	Significant delay in early VAP onset; shortens duration of mechanical ventilation and ICU stay
**Interleukin-7 (IL-7)**	Systemic lymphopenia (CIDS)	Expands lymphoid lineage (CD4+, NK cells) and rescues peripheral lymphocyte pool	Compassionate-use evaluations in severely immunosuppressed ICU patients	Compassionate-use clinical evaluations	No (Severely immunosuppressed ICU patients)	Theoretical risk of inducing secondary cytokine storms (though clinical data shows it is largely avoided)	Safely reverses absolute lymphopenia; restores adaptive immune responses to clear bacterial colonization
**Beta-adrenergic blockade** *(e.g., Propranolol)*	Sympathetic storm & lymphocyte apoptosis	Competitively blocks β2-adrenergic receptors, actively interrupting Bim-mediated apoptosis cascade in lymphocytes	Growing support in clinical literature; often utilized for paroxysmal sympathetic hyperactivity management	Emerging clinical observations, pre-clinical *in vivo* models	Yes	Risk of inducing systemic hemodynamic instability (bradycardia, hypotension) during the acute resuscitation phase	Blunts hyperadrenergic state, preserves peripheral lymphocyte pool, and potentially mitigates downstream infection risk
**Probiotics/synbiotics**	Gut dysbiosis & translocation vector	Restores intestinal barrier tight junctions, competitively excludes pathogens, and boosts SCFA production	Recommended as adjunctive strategy, but clinical efficacy is highly strain-specific	Meta-analyses of RCTs	No (Mechanically ventilated critically ill patients)	Risk of *Lactobacillus* or *Bifidobacterium* bacteremia in severely immunocompromised patients with impaired gut motility	relative risk reduction in VAP incidence; modest reduction in mechanical ventilation days
**Selective digestive decontamination (SDD)**	Oropharyngeal & gut pathogen reservoir	Proactively eradicates opportunistic Gram-negative reservoirs prior to micro-aspiration and biofilm formation	Routine use remains highly controversial despite evidence of early VAP reduction	Meta-analyses, Clinical trials	Yes (Trials exist specifically in severe neurological injury cohorts)	Substantial long-term risk of accelerating systemic antimicrobial resistance and multi-drug-resistant strains across the ICU	Significantly reduces the incidence of microbiologically confirmed early-onset pneumonia

### 5.4 Critical safety considerations

The clinical deployment of targeted immune-microbiome interventions carries inherent risks that must be carefully managed. The administration of IFN-γ, while mechanically capable of reversing macrophage paralysis, poses a severe safety risk if given indiscriminately during the acute, active phase of neuroinflammation. Without precision biomarker guidance, it may inadvertently exacerbate intracranial inflammatory states and worsen secondary brain injury. Similarly, probiotic administration, though beneficial for gut-lung barrier restoration, carries a risk of inducing Lactobacillus or Bifidobacterium bacteremia. This risk is particularly pronounced in severely immunocompromised neuro-ICU patients with profoundly impaired gastrointestinal motility or disrupted intestinal mucosal integrity. Finally, the routine use of SDD remains highly controversial due to long-term ecological implications; prolonged prophylactic antibiotic exposure in long-stay neuro-ICU patients carries a substantial risk of driving the emergence and dissemination of multi drug resistant antimicrobial strains.

## 6 Limitations

This narrative review possesses several important limitations. First, the foundational evidence supporting the proposed Brain-Lung-Immune-Microbiome axis, particularly regarding quantitatively significant hematogenous gut-to-lung microbial translocation and inter-kingdom signaling, derives predominantly from murine models and *in vitro* studies, which require careful extrapolation to human pathophysiology. Second, data regarding the epigenetic scarring of alveolar macrophages and mHLA-DR downregulation are frequently inferred from heterogeneous, non-TBI intensive care populations. Third, there is currently an absence of prospective human studies directly and comprehensively validating the proposed tripartite axis in its entirety specifically within TBI populations. Fourth, the cited microbiome sequencing studies exhibit methodological heterogeneity, utilizing varied platforms, variable 16S rRNA regions, and different bioinformatic pipelines, which complicates definitive cross-study comparisons. Finally, there remains a notable absence of large, multi-center, TBI-specific randomized controlled trials validating the proposed immunomodulatory therapies for VAP prevention, underscoring that these interventions are not yet ready for routine clinical implementation.

## 7 Conclusion

**Figure 3:**
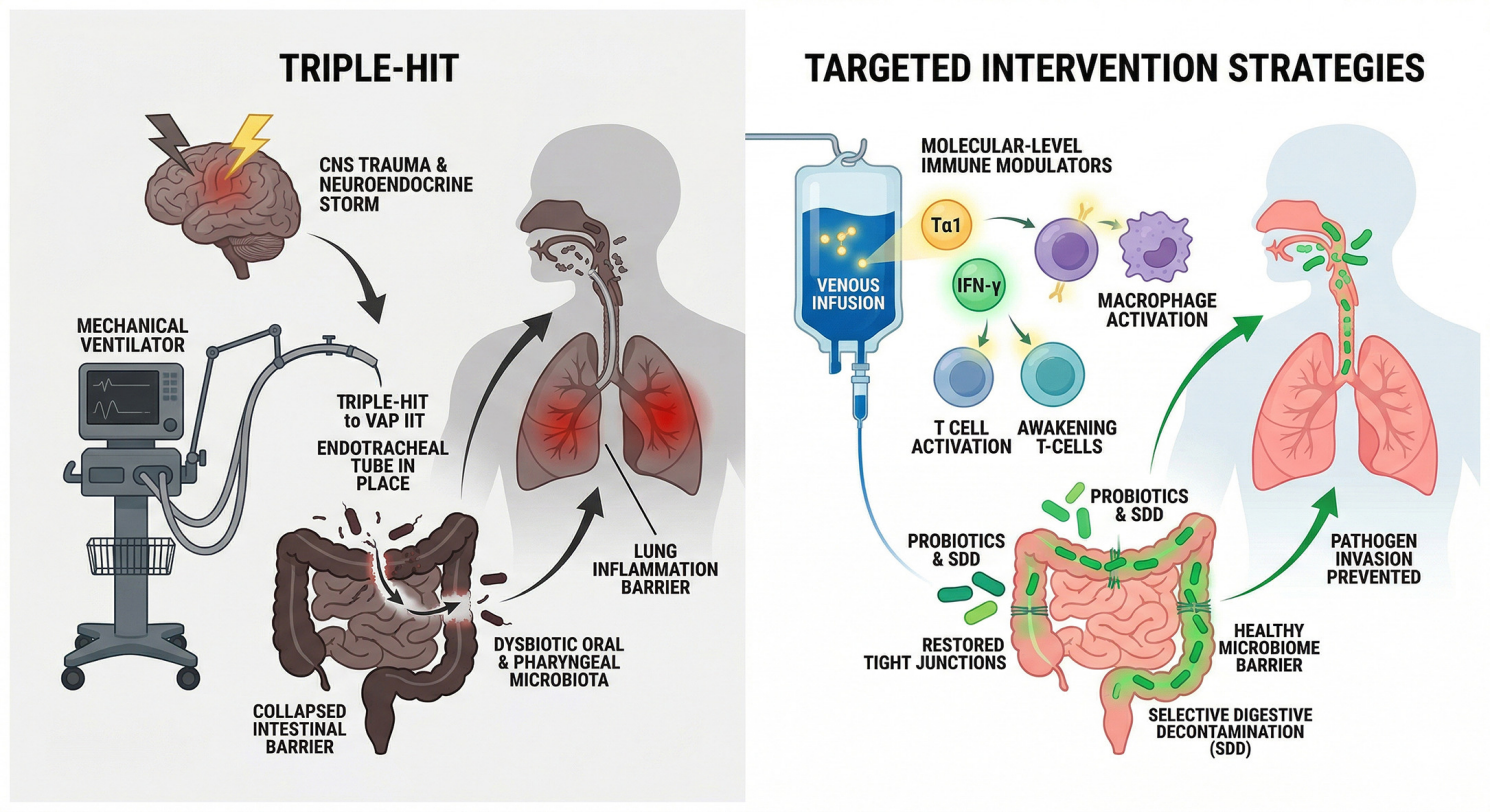
Pathological cascade and targeted intervention strategies based on the “triple-hit” hypothesis. The pathogenesis of ventilator-associated pneumonia in neurocritical care is driven by three sequential hits: neurogenic sympathetic hyperactivity, iatrogenic ventilatory injury, and systemic immune dysregulation coupled with intestinal dysbiosis. To counteract this cascade, targeted interventions focus on restoring immune-microbiome homeostasis. Host-directed immunomodulators, such as Thymosin α1 and Interferon-γ, aim to rescue apoptotic lymphocytes and reverse macrophage paralysis. Concurrently, microbiome-targeted therapies, including precision probiotics and SDD, are utilized to repair the intestinal barrier, prevent pathogen translocation, and maintain eubiosis.

The disproportionately high incidence, extreme morbidity, and significant mortality associated with ventilator-associated pneumonia in patients suffering from severe traumatic brain injury cannot be fully mitigated by standard, mechanically focused infection control bundles. To meaningfully advance outcomes in neurocritical care, the clinical and scientific perspective must rapidly expand from observing the lungs merely as isolated mechanical bellows subject to aspiration, to understanding them as integral, highly sensitive components of the complex Brain-Lung-Immune-Microbiome Axis (Figure 3).

The pathogenesis of VAP in this uniquely vulnerable population is the ultimate culmination of a catastrophic physiological domino effect. The initial, devastating neurological trauma instantly triggers a violent sympathetic and neuroendocrine storm. This massive chemical release directly induces profound systemic immune paralysis, characterized by the widespread, catecholamine-driven apoptosis of systemic lymphocytes and the deep, epigenetic deactivation of lung-resident alveolar macrophages. Deprived of normal immune surveillance and crippled by autonomic mucociliary stasis, the respiratory tract becomes highly vulnerable. Concurrently, the systemic shock wave disrupts the fragile intestinal barrier, causing a rapid, severe dysbiosis that translocate virulent enteric pathogens directly to the lungs via the bloodstream. In a final, remarkable display of biological complexity and inter-kingdom signaling, these invading, opportunistic pathogens actively exploit the host's surging stress catecholamines to aggressively construct drug-resistant biofilms, significantly reducing the efficacy of standard therapies.

Addressing this total, multi-system failure requires an urgent departure from rigid, one-size-fits-all antibiotic and ventilation protocols, moving purposefully toward dynamic, personalized, biologically integrated medicine. A promising future direction of VAP prevention and management in neuro-trauma lies in the real-time biomarker monitoring of immune status (such as serial mHLA-DR quantification) to guide the precise, safe administration of powerful immunomodulators like Thymosin alpha-1 or targeted IFN-γ. Furthermore, recognizing the systemic microbiome as a critical, highly functional organ necessitates the early integration of precision probiotics and targeted digestive decontamination to maintain eubiosis and prevent pathogen translocation. By simultaneously rearming the host's exhausted immune system and stabilizing the volatile microbial ecosystem, clinicians may potentially attenuate the pathological progression of the brain-lung axis, ultimately improve survival and maximize long-term neurological recovery in one of the most vulnerable critically ill populations in modern medicine.
